# Brain Activation of Patients With Obsessive-Compulsive Disorder During a Mental Rotation Task: A Functional MRI Study

**DOI:** 10.3389/fpsyt.2021.659121

**Published:** 2021-05-07

**Authors:** Sanghoon Oh, Wi Hoon Jung, Taekwan Kim, Geumsook Shim, Jun Soo Kwon

**Affiliations:** ^1^Department of Psychiatry, Seoul National University College of Medicine, Seoul, South Korea; ^2^Department of Psychiatry, Uijeongbu Eulji Medical Center, Eulji University School of Medicine, Gyeonggi-do, South Korea; ^3^Department of Psychology, Daegu University, Gyeongsan, South Korea; ^4^Department of Brain and Cognitive Sciences, Seoul National University College of Natural Sciences, Seoul, South Korea; ^5^KAIST Clinic Pappalardo Center, KAIST, Daejeon, South Korea; ^6^Institute of Human Behavioral Medicine, SNU-MRC, Seoul, South Korea

**Keywords:** obsessive-compulsive disorder, functional MRI, mental rotation task, visuospatial function, dorsolateral prefrontal cortex

## Abstract

Functional neuroimaging studies have implicated alterations in frontostriatal and frontoparietal circuits in obsessive-compulsive disorder (OCD) during various tasks. To date, however, brain activation for visuospatial function in conjunction with symptoms in OCD has not been comprehensively evaluated. To elucidate the relationship between neural activity, cognitive function, and obsessive-compulsive symptoms, we investigated regional brain activation during the performance of a visuospatial task in patients with OCD using functional magnetic resonance imaging (fMRI). Seventeen medication-free patients with OCD and 21 age-, sex-, and IQ-matched healthy controls participated in this study. Functional magnetic resonance imaging data were obtained while the subjects performed a mental rotation (MR) task. Brain activation during the task was compared between the two groups using a two-sample *t*-test. Voxel-wise whole-brain multiple regression analyses were also performed to examine the relationship between obsessive-compulsive symptom severity and neural activity during the task. The two groups did not differ in MR task performance. Both groups also showed similar task-related activation patterns in frontoparietal regions with no significant differences. Activation in the right dorsolateral prefrontal cortex in patients with OCD during the MR task was positively associated with their total Yale-Brown Obsessive-Compulsive Scale (Y-BOCS) scores. This study identified the specific brain areas associated with the interaction between symptom severity and visuospatial cognitive function during an MR task in medication-free patients with OCD. These findings may serve as potential neuromodulation targets for OCD treatment.

## Introduction

Obsessive-compulsive disorder (OCD) is a disabling neuropsychiatric condition characterized by recurrent, unwanted thoughts (obsession) and/or repetitive behaviors (compulsion), with a prevalence of 2–3% (1). Accumulated evidence from neuropsychological and neuroimaging studies in OCD has consistently demonstrated impairments in executive cognitive functions, such as inhibitory control and planning (2–4), and in the related neurocircuitry in the frontal-subcortical areas, called the cortico-striato-thalamo-cortical (CSTC) circuits (5–7). Recent studies also suggested the involvement of frontoparietal regions associated with working memory, particularly non-verbal memory, and visuospatial processing in OCD, although the involvement was rather inconsistent across studies (7–9). In addition, some evidence indicates that dysconnectivity of the frontoparietal network might have an important role in underpinning OCD symptomatology (10, 11).

Mental rotation (MR) is the ability to rotate mental representations for two- and three-dimensional objects in the mind (12), and an MR task is one of the well-known tasks for measuring visuospatial function (13, 14). Many studies of neuropsychological functions in OCD have documented deficits in visuospatial abilities (15), but the results of the MR task have not been consistent. Several studies have reported that patients with OCD are impaired significantly in MR ability compared with healthy controls (HCs) (16, 17). However, Moritz et al. (18) found no group difference in MR task performance when subjects were asked not only to count the sides of three-dimensional figures that were displayed but also sides that were not visible. Their subsequent study (9) also showed that OCD patients had no significant impairment in MR task performance compared to anxiety patients and HCs. It seems possible that these conflicting results are due to variations in study design, task condition, and task difficulty. Therefore, it would be helpful to implement an MR paradigm that has been well-validated and widely used (19).

The brain regions that are activated during an MR task are the superior parietal, frontal, and inferior temporal cortices (20). The posterior parietal/occipital cortex integrates visual and somatosensory information and is involved in implementing maps of space that code the locations of the targets of intended actions (21). Hence, these brain regions are suggested to be the main regions mediating MR performance (22). Performance on the MR task also requires the involvement of working memory, which recruits prefrontal areas such as the dorsolateral prefrontal cortex (DLPFC) and anterior cingulate gyrus (23). Neuroimaging studies employing MR tasks have provided a valuable means to elucidate the behavior of frontoparietal circuits underlying spatial working memory processes during MR in those with mental disorders. For example, Silk et al. (24) investigated alterations in brain activation in adolescents with attention-deficit hyperactivity disorder (ADHD) during an MR task and found that the ADHD group showed less activation in the “action-attentional” system (including Brodmann areas (BAs) 46, 39, and 40: the DLPFC and inferior parietal areas) and the superior parietal and middle frontal regions, suggesting a model of dysfunction of frontoparietal networks in ADHD. To date, however, no studies have investigated the functional neural correlates of MR in patients with OCD.

Therefore, the present study aimed to investigate the functioning of frontoparietal networks related to visuospatial abilities in medication-free patients with OCD. We first compared the performance of the MR task in OCD patients with HCs. Then, we used functional MRI (fMRI) to examine whether brain activation patterns during the MR task differed between OCD patients and HCs. In addition, the association of neural activation during the MR task with patients' symptoms was investigated to reveal the relationship between symptoms, cognitive function, and brain activity in OCD patients. Also, we hypothesized that a certain region belonging to frontoparietal circuits of OCD patients would be associated with obsessive-compulsive symptoms.

## Materials and Methods

### Participants

We recruited 26 medication-free patients with OCD (9 drug-naïve patients and 17 patients who were drug-free for at least 4 weeks) from the OCD clinic at Seoul National University Hospital. All patients underwent the Structured Clinical Interview for DSM-IV Axis I Disorders (SCID-I) and fulfilled the criteria for OCD. Twenty-six HCs matched for sex, age, and IQ were also recruited from the community. For all participants, the exclusion criteria were a lifetime history of psychosis, substance abuse/dependence, Tourette's disorder, or other tic-related conditions as well as a history of seizure disorder, head injury, or intellectual disability. Patients were assessed with the Yale-Brown Obsessive-Compulsive Scale (Y-BOCS) (25), the Hamilton Rating Scale for Depression (HAM-D) (26), and the Hamilton Anxiety Rating Scale (HAM-A) (27) to measure the severity of their obsessive-compulsive symptomatology, depression, and anxiety, respectively. From each participant, we collected T1-weighted high-resolution anatomical MRI, resting-state fMRI, diffusion tensor imaging, and fMRI scans during the MR task and the Tower of London (ToL) task. In this study, we focused on neural mechanisms associated with visuospatial ability using MR task-based fMRI data. Resting-state fMRI and ToL task-based fMRI data from this dataset were previously reported elsewhere (28, 29).

After pre-processing the fMRI data, 3 patients and 2 HCs were excluded due to excessive head motion [>2.5 mm of translation or 2.5° of rotation and >0.30 mm for mean framewise displacement (FD)] (30). One HC was also excluded due to missing data, and 8 subjects (OCD = 6, HCs = 2) dropped out after inclusion. Therefore, the final sample consisted of 17 patients with OCD (7 drug-naïve and 10 unmedicated for at least 4 weeks) and 21 matched HCs. Eight patients were free of Axis I or Axis II psychiatric comorbid disorders, and 9 had the following axis I psychiatric comorbidities: dysthymic disorder (*n* = 3) and depressive disorder not otherwise specified (*n* = 6). This study was approved by the Institutional Review Board of Seoul National University Hospital, and all participants signed an informed consent form prior to their participation. The demographic and clinical information for both groups is provided in Table 1.

**Table 1:** Demographic and clinical characteristics of patients with obsessive-compulsive disorder (OCD) and healthy controls (HCs).

**Variables**	**OCD patients**	**HCs**	***p-*value**
	**(*n* = 17)**	**(*n* = 21)**	
**Demographic characteristics**			
Age (years)	26.41 ± 5.98	26.00 ± 5.29	0.832^a^
IQ	112.41 ± 9.63	113.52 ± 10.80	0.742^a^
Sex (male/female)	12/5	11/10	0.254^b^
Handedness (left/right)	1/16	3/18	0.401^b^
**Clinical characteristics**			
Age of onset (years)	16.59 ± 6.03	–	
Duration of illness (years)	9.85 ± 6.85	–	
Y-BOCS scores			
Total	30.35 ± 4.14	–	
Obsession	16.18 ± 2.10	–	
Compulsion	14.18 ± 2.43	–	
HAM-D score	11.47 ± 6.17	–	
HAM-A score	12.41 ± 8.07	–	

*Data are presented as the mean ± SD or n. IQ, intelligence quotient; Y-BOCS, Yale-Brown Obsessive-Compulsive Scale; HAM-D, Hamilton Rating Scale for Depression; HAM-A, Hamilton Anxiety Rating Scale*.

a *Independent t-test or Welch's t-test if the variances were not equal*.

b *χ^2^ analysis or Fisher's exact-test for categorical data*.

### Mental Rotation Task

**Figure 1:**
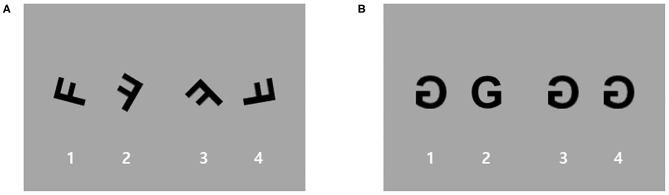
An example of stimuli used for the mental rotation (MR) task. Participants performed a block-designed MR task with two conditions: **(A)** an MR condition and **(B)** a zero rotation (ZR) condition as a control condition. For the MR condition, stimuli were rotated by four different angles (40, 80, 120, and 160 degrees), and these rotated characters were mirrored. For the ZR condition, only the characters generated by mirroring the image along the horizontal plane of the picture without any rotation (0 degrees) were used.

Participants performed a block-designed MR task with the following two conditions: (i) the MR condition and (ii) the zero rotation (ZR) condition as a control condition (Figure 1). For both the MR and ZR conditions, stimuli were three letters and three numbers (F, G, R, 2, 4, and 5). Because these alphanumeric stimuli are highly familiar that are usually displayed in an upright position, rotated characters are especially suitable for automatically triggering MR (31). The task consisted of four MR blocks and two ZR blocks. Stimuli in each block were presented pseudorandomly. Each block included 9 trials and was divided into resting fixation blocks of 20 s. The task was self-paced, with instructions to complete the task as quickly as possible without sacrificing speed for accuracy. In each trial, participants saw four rotated and/or mirrored characters (three identical mirrored characters and a different mirrored one) on the screen. Participants had to determine which of the alphanumeric characters was different from the others in each trial. For the MR condition, stimuli were rotated by four different angles (40, 80, 120, and 160 degrees), and these rotated characters were mirrored. For the ZR condition, only the characters generated by mirroring the image along the horizontal plane of the picture were used without any rotation (0 degrees). Before scanning, the participant practiced the task to learn the rules.

### Image Acquisition

Image data were acquired with a 3T scanner (Siemens Magnetom Trio, Erlangen, Germany) using a T2^*^-weighted gradient echo-planar imaging (EPI) sequence during the task. The parameters were echo time (TE) = 30 ms, repetition time (TR) = 2 s, flip angle (FA) = 90°, voxel size = 3.4 × 3.4 × 4.0 mm^3^, interleaved axial slices = 27, and number of volumes = ~360 per subject. High-resolution T1-weighted magnetization-prepared rapid-gradient echo anatomical images were obtained (TE = 1.89 ms, TR = 1.670 ms, FA = 9°, voxel size = 1.0 × 0.98 × 0.98 mm^3^, and sagittal slices = 208). To minimize possible motion artifacts, head cushions were used, and the participants were asked to move as little as possible during the acquisition.

### Statistical Analysis

Comparisons between OCD patients and HCs for demographic characteristics and behavioral performance during the MR task were made with independent *t*-tests or Mann-Whitney *U*-tests. A chi-square analysis or Fisher's exact-test was used for categorical data.

Image pre-processing and statistical analyses were performed using SPM12 software (http://www.fil.ion.ucl.ac.uk/spm). After discarding the first 4 volumes, data were corrected for both slice timing acquisition and head motion and spatially normalized to the Montreal Neurological Institute (MNI) reference brain. Then, data were smoothed with a 6-mm full-width at half-maximum (FWHM) Gaussian kernel.

For the first-level analysis, two regressors were generated by modeling the neural response to each of the MR and ZR conditions as a box car with an onset cycle equal to the length of each block, convolved with a hemodynamic response function. We computed the contrasts for the MR condition vs. the resting fixation condition and for the MR condition and ZR (control) condition for each subject. Next, these first-level contrast images were entered into second-level random effect analysis. One-sample *t*-tests were used for the patients and control groups separately to identify regions of significant activation during the MR task compared with the ZR and fixation condition. Two-sample *t*-tests were used to identify areas showing significant differences in activation in patients with OCD compared with HCs.

Voxel-wise whole-brain multiple regression analyses were also performed to examine whether there was an association between patient symptom severity (total Y-BOCS score) and neural activation during the MR task. Patients' HAM-D scores were included in the model as a covariate to control for their depressive symptoms. All statistical results were set at a cluster-level threshold of *p* < 0.05, family-wise error (FWE) corrected for multiple comparisons, and a voxel-level threshold of *p* < 0.001, uncorrected.

## Results

### Behavioral Data

Table 2 shows behavioral performance, including response times, accuracy, and efficiency (defined by the accuracy divided by the response time), for each group during the MR task and the statistical results. No significant between-group difference was observed for any of the performance variables. There was a trend, however, for OCD patients to show slower responses and increased error rates than HCs during the task.

**Table 2:** Behavioral performance during the mental rotation task in patients with obsessive-compulsive disorder (OCD) and healthy controls (HCs).

	**OCD patients**	**HCs**	**Statistical analysis**^**a**^	
	**(*n* = 17)**	**(*n* = 21)**	***Z***	***p*-value**
Response time (RT)^b^	6.29, 1.91	5.24, 0.93	−1.763	0.078
Accuracy^c^	83.48, 16.56	90.86, 7.41	−1.350	0.177
Efficiency^d^	15.00, 6.41	17.95, 3.87	−1.688	0.091

*Data are presented as the mean ± SD*.

a *Mann-Whitney U-test for testing the group difference*.

b *Average RT of correct responses for the mental rotation condition, in seconds*.

c *Percentage (%) of correct responses for the mental rotation condition*.

d *Defined as the accuracy divided by the response time*.

### Neuroimaging Data

**Figure 2:**
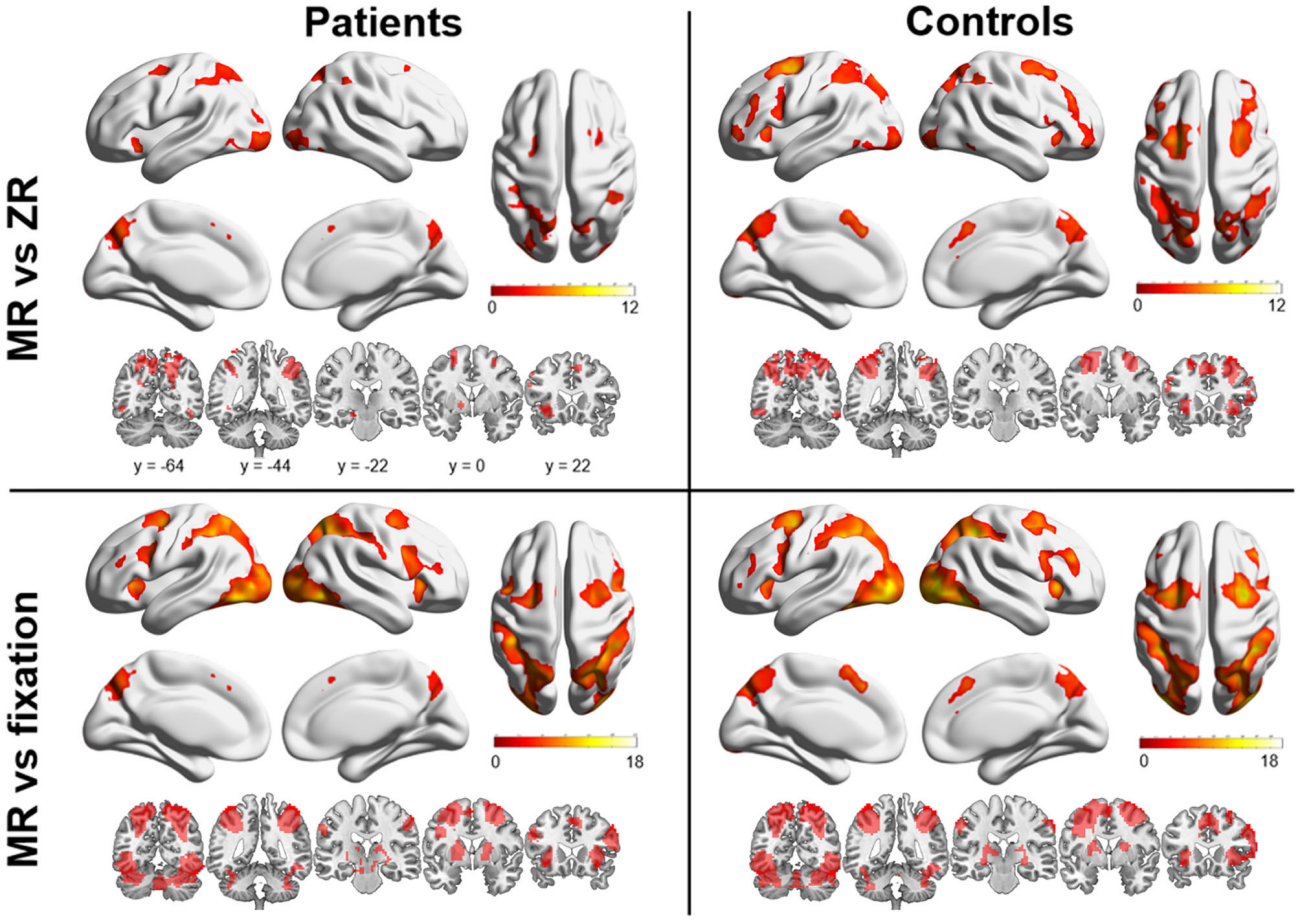
Brain regions are shown that were significantly activated in patients with OCD and healthy controls (HCs) during the mental rotation (MR) condition vs. the zero rotation (ZR) condition and in the MR condition vs. the fixation condition. Markedly increased activation was observed in the frontal and parietal areas in both groups.

Figure 2 shows the main effect of the MR condition vs. fixation (or ZR) condition for each group. In a comparison of the MR condition and the fixation condition, both groups exhibited bilateral activation in the parietal lobe, predominantly in the superior and inferior areas, extending into the occipital lobe. Activation was also found in a considerable portion of the bilateral frontal areas, including the superior frontal gyrus, middle frontal gyrus, and superior medial frontal gyrus, and subcortical areas, including the striatum and thalamus. A comparison between the MR condition and the ZR condition revealed an overall similar activation pattern to that observed in the comparison between the MR condition and the fixation condition. Both groups showed bilateral activation in a considerable portion of the parietal and frontal areas. However, few activations in subcortical regions were observed. On the other hand, between-group analysis did not show any significant differences in brain activation during the MR task between patients with OCD and HCs.

**Figure 3:**
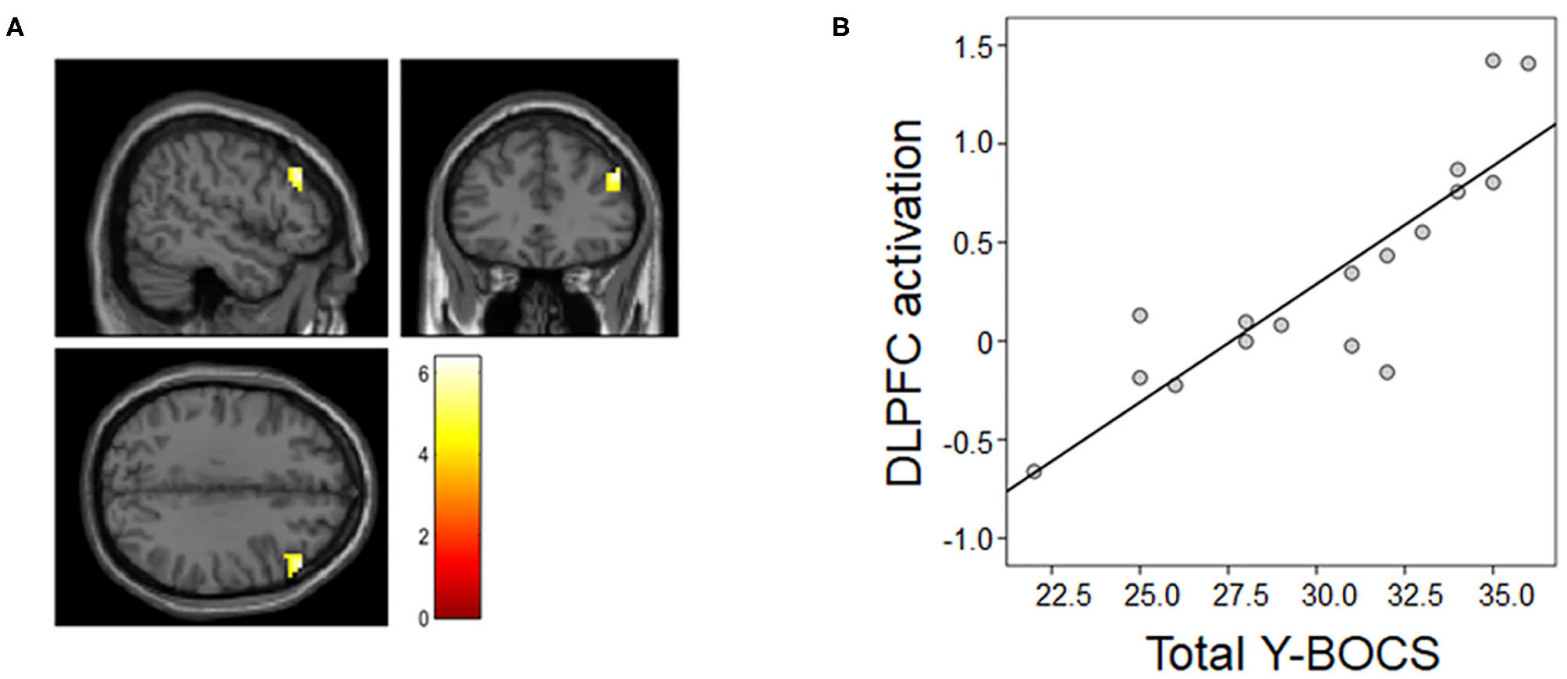
Association between neural activation during the MR task and symptom severity. **(A)** Patients' total Y-BOCS scores were associated with right dorsolateral prefrontal cortex (DLPFC) activation in the mental rotation (MR) condition vs. the zero rotation (ZR) condition. **(B)** For illustration purposes, we extracted the magnitude of DLPFC activation showing the above association and then assessed Pearson's correlation between the magnitude of activation and the total Y-BOCS score.

The multiple regression analysis results are presented in Figure 3. The voxel-wise whole-brain multiple regression analysis revealed a significant positive association between the contrast images of the MR condition vs. the ZR condition and clinical symptom severity in OCD patients, showing that the more severe the obsessive-compulsive symptoms were, the greater the activation was in the right DLPFC (*x, y, z* = 51, 33, 33; *t*-/*z*-values = 6.39/4.30; cluster size = 75 voxels) of patients.

## Discussion

To the best of our knowledge, the present study was the first to evaluate the neural responses of patients with OCD while they were performing an MR task. Particularly, all patients were medication-free at the time of the MRI scan. We found that frontoparietal regions showed significant activation during the MR task in both OCD patients and HCs, but there were no significant group differences in brain activation. A conspicuous finding from our data was that higher clinical symptom severity in patients with OCD was significantly associated with greater activation in the right DLPFC. Therefore, these findings suggest that the frontoparietal network is implicated in visuospatial cognition, and the DLPFC in this network is an important element in the pathophysiology of OCD.

The brain circuits activated during the MR task were almost the same in patients and HCs and included the bilateral parietal, occipital, and frontal regions. These regions have been reported consistently in previous neuroimaging studies of MR (20). This finding further supports the idea that MR is a form of spatial working memory task that activates frontoparietal networks (23). Specifically, activation of the superior parietal cortex and adjacent regions appears to reflect visuospatial processing components of MR (23, 32, 33), and the activation of motor regions in the pre-central cortex implies the use of motor stimulation to solve MR problems (20, 34). The additional activation of prefrontal areas may reflect the role of working memory in MR (22, 35).

However, we were unable to identify any regions showing significantly increased or decreased activation in OCD patients compared with HCs during the MR task. This result may be explained by the fact that the patients demonstrated a level of performance on the MR task that was similar to that of the control subjects. Likewise, Nakao et al. (36) found that the patients with OCD and normal controls did not differ in their performance on neuropsychological tests and showed similar brain activation on fMRI during the Stroop test. Given the evidence of impaired visuospatial ability in patients with OCD (37), it is somewhat surprising that there were no significant group differences in MR task performance. Several factors could be attributed to this finding. Because of their comparably high intelligence, OCD patients may have compensated for suboptimal behavioral performance (38, 39). A number of studies have reported sex differences in the MR task, with men performing better than women (40–42). Our study included a slightly larger proportion of males in the patient group relative to control subjects (although there were no significant differences in demographic characteristics), possibly resulting in an overestimation of the performance scores on average in the OCD group. Furthermore, the low difficulty of the MR task that we used in the present study may have masked subtle behavioral or neurocognitive differences between the two groups. We used simple two-dimensional alphanumeric stimuli with relatively low difficulty rather than a two-dimensional representation of tri-dimensional figures (17). Indeed, it has been observed in neuropsychological studies that OCD patients show normal cognitive performance on low-demand cognitive tasks but show cognitive deficits at tasks with higher cognitive demands (43, 44). Although the task consisting of rotated alphanumeric stimuli properly activates the frontoparietal areas subserving MR (22, 31), further studies using more adequate task conditions that can generate cognitive load sufficiently are suggested.

The most important clinically relevant finding was that neural activation in the right DLPFC during the MR task was positively associated with obsessive-compulsive symptom severity. Previous studies suggested that there was a relationship between the orbitofrontal-striatal circuit and the generation or severity of obsessive-compulsive symptoms (45, 46). In addition to dysfunction of this circuitry, there has been considerable recent evidence that the frontoparietal networks, including the DLPFC and parietal regions, are also affected in OCD (5, 7). Indeed, de Vries et al. (47) found that OCD patients showed task-related hyperactivation in the left dorsal frontal areas and left precuneus associated with better task performance. Moon and Jeong (48) reported that a BOLD signal change in the DLPFC of patients with OCD during face-recognition tasks was negatively correlated with their Y-BOCS scores. In prior resting-state fMRI studies, altered connectivity regarding the DLPFC has been found in OCD patients relative to HCs, suggesting that the DLPFC might be central to OCD pathophysiology (11, 49). A more recent rs-fMRI study using bivariate Granger causality analysis demonstrated the abnormal causal interactions of DLPFC-related circuits in patients with OCD (50). Our finding that greater activation in the DLPFC during the MR task was associated with severe obsessive-compulsive symptoms may be related to patients' efforts to resist their symptoms during cognitive tasks. This idea is supported by data that the DLPFC is activated when patients with OCD inhibit their obsessive processes (5) or compulsive behaviors (51). Furthermore, it has been reported that the DLPFC is directly connected with the frontostriatal circuit of OCD patients (52) and is important in the integration of emotion and cognition (53). These roles and abnormalities of the DLPFC have made it an attractive target for neuromodulation. Indeed, a recent meta-analysis of randomized controlled trials demonstrated that repetitive transcranial magnetic stimulation (rTMS) targeting the DLPFC yielded greater improvements in obsessive-compulsive symptoms than sham rTMS (54). Taken together, our finding of the association between visuospatial task-related activation in the DLPFC and obsessive-compulsive symptom severity seems to be consistent with these earlier findings. This finding also provides new evidence of the involvement of abnormalities in the frontoparietal circuits in the pathophysiology of OCD.

Several limitations to this study need to be acknowledged. First, the sample size in the final analysis was relatively small. Some participants were excluded due to the quality of the functional imaging data, and the decreased number of subjects might have affected the statistical power of the results. Therefore, future studies with larger sample sizes are needed to confirm the findings of the present study. Second, nine of the 17 patients with OCD had comorbid axis I disorders such as dysthymic disorder or depressive disorder not otherwise specified. Given that depressive symptoms influence task performance and decreased performance is related to imaging results (18), it is important to bear clinical characteristics in mind when interpreting these results. Third, no information was collected about what participants subjectively experienced (e.g., whether they felt distressed, irritated, or compulsive) during the scan. These emotions may have affected their cognitive task performance and/or neural activity (55). We should also consider the learning effect on the MR task because all subjects practiced the task prior to the fMRI scan. Last, our patient sample consisted largely of males, and sex has been suggested to contribute to the biological heterogeneity of OCD (56); thus, this high proportion of males in our sample limits the generalization of our findings.

## Conclusions

In conclusion, this fMRI study provides the first evidence that neural activation in unmedicated patients with OCD when performing an MR task is not different from that in HCs but is associated with obsessive-compulsive symptom severity. These results improve the present understanding of the neural mechanisms of MR associated with symptoms in OCD and thus provide the potential to identify neuromodulation targets for managing patients' symptoms.

## Data Availability Statement

The raw data supporting the conclusions of this article will be made available by the authors, without undue reservation.

## Ethics Statement

The studies involving human participants were reviewed and approved by the Institutional Review Board of Seoul National University Hospital. The patients/participants provided their written informed consent to participate in this study.

## Author Contributions

WJ, GS, and JK designed the study. WJ, TK, and GS contributed to the acquisition of MRI data. JK collected the clinical data, interpreted the data, and gave critical comments on the manuscript. SO and WJ performed data analysis and wrote the first manuscript. All authors critically reviewed the manuscript content and approved the final version for publication.

## Conflict of Interest

The authors declare that the research was conducted in the absence of any commercial or financial relationships that could be construed as a potential conflict of interest.
